# Reduced selection for antibiotic resistance in community context is maintained despite pressure by additional antibiotics

**DOI:** 10.1038/s43705-023-00262-4

**Published:** 2023-05-31

**Authors:** Peiju Fang, Alan Xavier Elena, Maxi Antonia Kunath, Thomas U. Berendonk, Uli Klümper

**Affiliations:** Technische Universität Dresden, Institute of Hydrobiology, Zellescher Weg 40, Dresden, Germany

**Keywords:** Water microbiology, Microbial ecology, Antibiotics

## Abstract

Selection for antibiotic resistance at very low antibiotic concentrations has been demonstrated for individual antibiotics in single species experiments. Furthermore, selection in these focal strains is reduced when taking place in complex microbial community context. However, in the environment, bacteria are rarely exposed to single, but rather complex mixtures of selective agents. Here, we explored how the presence of a second selective agent affects selection dynamics between isogenic pairs of focal *E. coli* strains, differing exclusively in a single resistance determinant, in the absence and presence of a model wastewater community across a gradient of antibiotics. An additional antibiotic that exclusively affects the model wastewater community, but to which the focal strains are resistant to, was chosen as the second selective agent. This allowed exploring how inhibition alters the community’s ability to reduce selection. In the presence of the community, the selection coefficient at specific antibiotic concentrations was consistently decreased compared to the absence of the community. While pressure through the second antibiotic significantly decreased the activity and diversity of the community, its ability to reduce selection was consistently maintained at levels comparable to those recorded in absence of the second antibiotic. This indicates that the observed effects of community context on selection dynamics are rather based on competitive or protective effects between the focal strains and a small proportion of bacteria within the community, than on general competition for nutrients. These findings have implications for our understanding of the evolution and selection for multi-drug resistant strains.

## Introduction

The discovery of antibiotics plays an important role in treating infections caused by bacteria and extending the average human lifespan. While antibiotic resistance is ancient, the use and overuse of antibiotics in recent decades has contributed to the emergence and spread of antibiotic resistance in human pathogens and the environment [1, 2]. Antibiotic resistance accounts for around 700,000 deaths annually and could exceed 10 million by 2050, hence antibiotic resistance has been recognized as a major global health threat by the World Health Organization [3, 4]. Aquatic environments which frequently receive antibiotic residues provide an ideal setting for the accumulation and dissemination of antibiotic resistance [5]. Previous studies demonstrated that the environment serves as an important source of antibiotic resistant bacteria (ARB) and antibiotic resistance genes (ARGs) and a hotpot for their spread, with human activities significantly enriching ARB and ARGs in the environment [6–11]. The problems associated with the spread of antibiotic resistance need to consequently be addressed in a global context and across the highly interconnected human, veterinary as well as environmental spheres within a “One Health” context [12]. To achieve this, the limited knowledge regarding the contribution of the environment on selection and maintenance of resistant bacteria needs to be extended [6, 13].

Antibiotic resistance emerges in the environment through evolutionary processes or anthropogenic introduction. It is then selected and maintained by the selective pressures posed by agents such as antibiotics, heavy metal and biocides [8, 14]. The maintenance of ARGs generally imposes fitness costs on the host bacteria, due to the metabolic burden connected to their expression [14, 15]. This in turn reduces the growth rate of resistant bacteria compared to their susceptible counterparts. However, these costs are negligible under selection pressure due to the benefits of being resistant [14, 15]. The minimal selective concentration (MSC) is defined as the lowest concentration of a corresponding antibiotic at which resistant strains are selectively favored over susceptible strains, while the minimum inhibitory concentration (MIC) refers to the lowest concentration at which the susceptible strains are completely inhibited by the antibiotic. Previous studies using competition experiments provided evidence that MSCs, above which positive selection for resistant bacteria occurs, are much lower than the MICs. For most antibiotics these MSCs even fall within the environmentally detected concentration range [16–18]. This highlights that antibiotic pollution even at very low concentrations could pose a risk on environmental and human health. However, these previous studies assessed MSCs based on the relative fitness for isogenic variants of focal strains, which only differ in a single ARG [16]. While competition experiments of susceptible and resistant focal strains reveal general information regarding the MSCs and fitness costs of ARGs, the environmental realism is low. To improve this experimental deficiency, Klümper et al. performed competition experiments of such focal strains while embedded in a complex microbial community, which led to a 1-2 orders of magnitude increase in the observed MSCs, resulting in severe effects on the outcome of competition [19]. The identified mechanisms underlying this increase in MSCs included an increase in the cost of resistance when in competition with other community members, as well as the potential protection of the susceptible focal strain through other, potentially resistant community members [19].

A crucial feature that is currently missing when assessing MSCs in environmental scenarios is that environmental pollution through for example municipal, hospital or pharmaceutical wastewaters is usually not limited to a single, but complex combinations of selective agents [20–22]. Exposure to a combination of different antibiotics is a particularly important scenario, as the evolution and selection of multidrug resistant (MDR) bacteria could be specifically favored [23]. MDR bacteria refer to those bacteria that are resistant to a broad range of antibiotics. Such MDR pathogens have been reported to cause high mortality rates worldwide, and pose a severe challenge towards human health [24, 25]. In addition to direct effects on selection for multidrug resistance, the presence of multiple antibiotics might affect the previously reported protective and competitive effects that occur in community context [19]. Complex communities contain different species and genotypes, which occupy different niches and have different functions [26]. Consequently, selectively inhibiting certain community members through pressure with additional antibiotics could lower their protective and competitive abilities.

Hence, we expect that selection for multidrug resistance in a focal species in the presence of a single antibiotic will be reduced in the presence of a community as determined earlier for single resistances [19]. However, we hypothesize that this effect could be alleviated or even completely abolished in the presence of additional antibiotics if community activity and functions, including potential protective effects of susceptible strains are reduced. Such effects would be highly dependent on the composition and resistance profile of the community and on if those community members that are particularly involved in competitive or protective interactions with the focal strain are indeed inhibited by the pressure through additional antibiotics.

To explore this, we created single and multidrug resistant strains of a focal *E. coli* species, differing exclusively in the introduced ARGs. We then pairwise competed these strains in the absence and presence of a model wastewater community across different concentrations and combinations of two antibiotics. To the first of these antibiotics only the multidrug resistant strain is resistant. To the second one both focal strains are resistant. Either of these two antibiotics, individually or in combination, have the potential to inhibit the complex community. In order to provide explanations for the observed results from these competition experiments, the effect of the antibiotics on the activity and composition of the wastewater community were explored in combination with the fitness of the focal strains.

## Material & methods

### Focal strains

The wild type of *Escherichia coli* MG1655 [27] was used to create the focal strains for competition experiments (Table 1). First, single resistant variants were created hosting either gentamicin or kanamycin resistance. The wild-type strain was chromosomally tagged with gentamicin resistance gene *aacC1*, or kanamycin resistance gene *aphA* respectively, through electroporation with pBAM delivery plasmids (pBAMD1-2 for kanamycin, pBAMD1-6 for gentamicin [28]) containing the mini-Tn*5* delivery system. Successful clones were screened for gentamicin (20 μg mL^−1^) or kanamycin (50 μg mL^−1^) resistance on LB agar plates. Further loss of the delivery plasmid was confirmed through susceptibility to ampicillin (100 µg mL^−1^). Finally, in the selected strains the inserts were confirmed to be not located in any coding regions based on whole genome sequencing of the respective strains and alignment with the *E. coli* MG1655 reference genome [27]. Whole genome sequences were stored in the NCBI Sequence Read Archive (SRA) database under project number PRJNA865074.

**Table 1:** List of strains created and used in this study.

Bacterium	Resistance gene	Resistant to	Reference
*E. coli* MG1655			[27]
*E. coli* MG1655	*aacC1*	Gentamicin	This study
*E. coli* MG1655	*aacC1*, *aphA*	Gentamicin, Kanamycin	This study
*E. coli* MG1655	*aphA*	Kanamycin	This study
*E. coli* MG1655	*aphA*, *aadA*	Kanamycin, Streptomycin	This study

Previous studies have demonstrated that the acquisition of resistance to one antibiotic could increase (positive cross-resistance) or decrease (negative cross-resistance or collateral sensitivity), the tolerance towards other antibiotics [29, 30]. Despite the fact that *aacC1* and *aphA* confer resistance to antibiotics of the same antibiotic class (aminoglycosides), no cross resistance of *aacC1* towards kanamycin and *aphA* towards gentamicin was observed based on tests of the maximum growth rate under antibiotic exposure at the concentrations used in the competition experiments (see below) when compared to the susceptible ancestral strain.

To create the multidrug resistant strains, the gentamicin resistant strain was further tagged with kanamycin resistance gene *aphA* (Kn^R^) through a second round of electroporation with the pBAM1-2 delivery plasmid [28]. Successful clones were screened for gentamicin (20 μg mL^−1^) and kanamycin (50 μg mL^−1^) resistance simultaneously on LB agar plates with the *aphA* insert in the gentamicin and kanamycin resistant strain (Gm^R^Kn^R^). Similarly, the kanamycin resistant strain was further tagged with the streptomycin resistance gene *aadA* through a second round of electroporation with the pBAMD1-4 delivery plasmid [28] and screened on LB Agar containing kanamycin (50 μg mL^−1^) and streptomycin (100 μg mL^−1^) to obtain the strain Kn^R^Sp^R^. Again, no cross resistance of *aadA* towards kanamycin and *aphA* towards streptomycin was observed under antibiotic exposure at the concentrations used in the competition experiments.

Ultimately, we obtained two pairs of strains that consisted of one single and one multidrug resistant strain, which were used to perform the competition experiments (Gm^R^ vs Gm^R^Kn^R^ and Kn^R^ vs Kn^R^Sp^R^) across different concentrations of antibiotics and in presence and absence of a model microbial community.

### Model wastewater community

As the model community, a wastewater microbial community was used throughout the experiments to provide the community context in which competition experiments between the focal pairs of strains could take place. The wastewater microbial community was collected from the effluent of the wastewater treatment plant Dresden-Kaditz, Germany (51.07 °N, 13.67 °E) in November 2020. In total, 20 L of effluent water were sampled in sterile plastic bottles, and immediately transported to the laboratory on ice. The microbial community in the 20 L effluent water was collected through centrifugation (20 min, 4 °C, 4 000 rpm, Eppendorf AG, Hamburg, Germany). The cell pellet was then resuspended in 200 mL 50% sterile glycerol solution containing 9 g L^−1^ NaCl, homogenized by vortexing and frozen at −80 °C in 1 mL aliquots of a 100× concentrated wastewater effluent community. This ensured that for every experiment in this study the identical wastewater community inoculum could be used.

### Growth medium

All experiments were carried out in a modified version of M9 minimal medium [31] which supported growth of all focal strains as well as the wastewater microbial community. Per liter 20 ml of 1 M sodium citrate dihydrate, 10 g of tryptone and 5 g of yeast extract were added as carbon sources.

### Maximum growth rate

The maximum growth rates of each of the strains as well as the wastewater community were obtained for each of the antibiotic concentrations and combinations mentioned above. To a sterilized 96 well plates, 3 µL of the bacterial culture adjusted to OD_600_ = 0.5 and 297 µL of fresh medium with the corresponding antibiotics were added in 6 replicates. Plates were then incubated at 37 °C with continuous shaking in a microplate reader (BioTek Synergy H1, Winooski, VT, USA). Optical density (OD) at 600 nm was measured every 10 min during a 24 h incubation period. The maximum growth rates (μ_max_) for each treatment were calculated based on these OD_600_ readings as the maximum slope during the exponential growth phase in the growth curve.

### Competition assays

For setting up the competition experiments the respective focal strains with the relevant antibiotics and the wastewater community in the absence of antibiotics (inoculated from the 100× concentrated freezer stock at 1:100 ratio) were grown separately in three 10 mL replicates each at 37 °C, 120 rpm for 24 h. Replicates were combined, harvested by centrifugation, washed twice in 0.9% NaCl and adjusted to OD_600_ = 0.5 for inoculation of the competition experiment. Competition experiments were carried out in 50 mL glass vials with 10 mL of growth medium. Vials were inoculated with a total of 100 µL of the bacterial solutions resulting in a final bacterial concentration of approximately 10^6^ bacteria mL^−1^. In the absence of the community the respective single and multidrug resistant strains were mixed at 1:1 ratio to obtain the inoculum. In the presence of the community the vials were inoculated with the two strains at 5 µL each and with 90 µL of the model community to achieve a final 1:10 ratio between focal strains and community, which was previously successfully used in such competition assays [19].

The pair of the single gentamicin (Gm^R^) and multidrug gentamicin kanamycin resistant (Gm^R^Kn^R^) strains was competed across three gentamicin (Gm) concentrations in combination with two kanamycin (Kn) concentrations: Gm 0, 5, 10 μg mL^−1^, Kn 0, 2.5 μg mL^−1^. The second pair consisting of the kanamycin resistant (Kn^R^) and kanamycin streptomycin resistant (Kn^R^Sp^R^) strains were competed across three kanamycin concentrations in combination with two streptomycin (Sp) concentrations: Kn 0, 12.5, 25 μg mL^−1^, Sp 0, 25 μg mL^−1^. Replicate reactors of each combination of antibiotic concentrations in the absence and presence of the community were set up and grown at 37 °C with 120 rpm shaking for 24 h (*n* = 3–6). After 24 h, 100 μL of each reactor were transferred to a new vial with 10 mL fresh medium and freshly added antibiotics. This transfer was performed twice, and reactors were harvested after the third and final 24 h growth cycle. A ten-fold serial dilution with 0.9% NaCl solution was performed for each reactor, and plated out on selective Chromocult coliform agar plates with corresponding antibiotics (Gm 20 μg mL^−1^, Gm 20 μg mL^−1^ + Kn 50 μg mL^−1^, Kn 50 μg mL^−1^, Kn 50 μg mL^−1^ + Sp 100 μg mL^−1^) to enumerate the violet *E. coli* colonies of the respective strains. The abundance of the single resistant strain was calculated based on the number of colonies on the single antibiotic plate minus those on the double antibiotic plate. Preliminary tests of only the wastewater community plated out on these selective plates confirmed that no resistant *E. coli* were present in the model community, which could otherwise interfere with the accuracy of the plate counts.

The Malthusian growth parameters and relative fitness were calculated according to Lenski et al. [32]. Briefly, the realized Malthusian parameter of each strain was initially defined (Eq. (1)):

1 $$mA_{t = i} = \frac{{ln\left( {A_{t = i}/A_{t = 0}} \right)}}{{t_i}}$$

where A_t=0_ refers to the density of strain A at time 0 (inoculation) and A_t=i_ refers to density of strain A at time i, here after 3 days. Then the relative fitness (W) of the resistant strain B compared to the susceptible strain A was calculated according to Eq. (2):

2 $$W_{t = i} = mB_{t = i}/mA_{t = i}$$

### Antibiotic degradation test

To explore the stability of the antibiotics during the competition experiments, we tested the inhibitory effects of the supernatant after 3 days of competition, compared to a supernatant of the antibiotic free controls spiked with fresh antibiotics of the same initial concentrations. We collected the supernatant of the competition experiment in the presence of the wastewater community without antibiotics and for the Gm 10 μg mL^−1^ + Kn 2.5 μg mL^−1^ treatment by centrifugation of 10 mL of cultures (10 min, 4 °C, 4 000 rpm) (*n* = 3). We similarly collected supernatant from the wastewater community growing in isolation (without the focal strains) in the absence of antibiotics and grown at Gm 10 μg mL^−1^ + Kn 2.5 μg mL^−1^ (*n* = 3). All supernatants were sterilized by filtration through 0.22 μm pore size membrane filters. Supernatants were supplemented in 1:10 ratio with 10× lysogeny broth to ensure fresh nutrition. Fresh antibiotics were added to the supernatants from the non-antibiotic treatment to also achieve final concentrations of Gm 10 μg mL^−1^ + Kn 2.5 μg mL^−1^. The susceptible focal strain was then grown in these supernatant media for 24 h, and the maximum growth rate was measured according to the OD_600_ based protocol mentioned above in 2 technical replicates for each of the 3 biological replicates of each supernatant mixture.

### DNA extraction, 16S rRNA high-throughput sequencing and bioinformatic analysis

To gain insights into the evolution of community composition in the competition experiments between the Gm^R^ and Gm^R^Kn^R^ strain in the presence of the community, we performed 16S rRNA gene-based sequencing. Two mL from each replicate vial at each treatment of the competition experiment between the Gm^R^ and Gm^R^Kn^R^ strain in the presence of the wastewater community was harvested by centrifugation at the end of the competition experiment. Similarly, 2 mL of the initial wastewater community inoculum (3 replicates) were harvested. DNA was extracted using the Qiagen PowerSoil kit following the manufacturer’s instructions. DNA quality and concentration were confirmed by NanoDrop 2000 (Thermo Fisher Scientific, Waltham, MA, USA). The hypervariable regions V3-V4 of the 16S rRNA gene were amplified using the PCR primers 341F and 806R [33, 34]. The PCR products were further purified, quantified and sequenced on the Illumina MiSeq platform (Illumina Inc., San Diego, CA, USA) at Eurofins Genomics Germany GmbH (Ebersberg, Germany). The sequencing analysis was performed in MOTHUR v 1.48.0 [35], according to the SOP [36]: operational taxonomic units (OTUs) were clustered at 97% similarity level and annotated based on SILVA v138 [37]. All chimeras, chloroplast, mitochondrial, archaeal, eukaryotic and unknown sequences were removed. Sequencing libraries were subsampled to 30 000 sequences per sample and these final sequences were clustered into 1987 OTUs. The raw sequences were stored in the NCBI Sequence Read Archive (SRA) database under project number PRJNA865074.

In order to predict if aminoglycoside ARGs should be expected in one of the most dominant species observed in the wastewater community, *Myroides* spp., complete genomes of 17 *Myroides* isolates were downloaded from NCBI Genbank (Supplementary Table S1). Antimicrobial resistance genes were screened in each retrieved genome using AbritAMR [38] and the Resfinder (v. 24/05/2022) and AMRFinder [39] databases separately.

### Statistics

The diversity index and Bray–Curtis similarity were calculated in R v4.2.0 using the “vegan” package [40]. Pearson’s correlation coefficients between Chao 1 and antibiotic concentrations were calculated in SPSS v22.0 (IBM Corp, Armonk, NY, USA). Non-metric multidimensional scaling ordination (NMDS) and analysis of similarity (ANOSIM) were performed in PRIMER v7.0 [41], based on Bray–Curtis similarity. One-way analysis of variance (ANOVA) was performed in SPSS v22.0, and significant differences (*P* < 0.05) within groups were calculated by Tukey and Dunnett’s T3 post hoc test based on the homogeneity of the data set.

## Results

### Effects of antibiotic exposure on selection in absence of the community

**Fig. 1:**
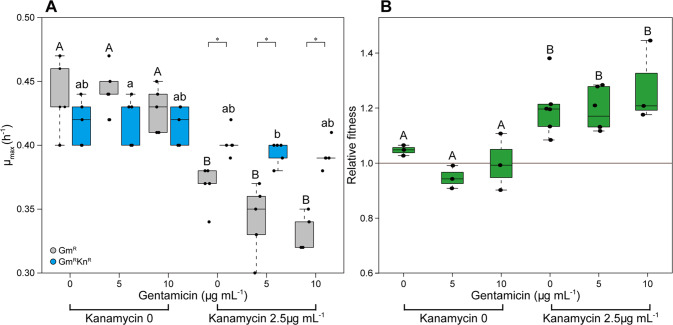
Kanamycin decreased the activity of the Gm^R^ strain. **A** Maximum growth rate per hour of the Gm^R^ and the Gm^R^Kn^R^ strain across the gradient of gentamicin and kanamycin concentrations. **B** Relative fitness of the Gm^R^Kn^R^ strain vs Gm^R^ strain. Values are mean ± standard deviation. Significant differences between groups are indicated with stars. * = *P* < 0.05. Significant differences within groups are indicated with different letters (*P* < 0.05), there were no significant differences between the samples that have the same letters, uppercase and lowercase represent different groups.

To determine the fitness effects of the antibiotics on the focal strains, the isogenic *E. coli* strains with single (Gm^R^) and multidrug resistance (Gm^R^Kn^R^) were first grown individually and then directly competed across the combinations of two kanamycin and three gentamicin concentrations. In the individual growth rate assay, the maximum growth rate of the exclusively Gm^R^ strain was significantly lower in the presence of kanamycin at 2.5 μg mL^−1^ than in the absence of kanamycin by 20.2 ± 5.7% (*P* < 0.05, mean ± SD, ANOVA) (Fig. 1A). Contrary, kanamycin had no effect on the maximum growth rate of the Gm^R^Kn^R^ strain (*P* = 0.06) (Fig. 1A). Gentamicin, which both strains possess resistance to, had no significant effect on the maximum growth rate of either strain (Gm^R^, Gm^R^Kn^R^) independent of the presence and absence of kanamycin. In each of these four scenarios the growth rate at 0 μg mL^−1^ displayed no significant difference to that at 10 μg mL^−1^ of gentamicin based on ANOVA tests with Tukey and Dunnett’s T3 post hoc test (all *P* > 0.05) (Fig. 1A).

In competition experiments between the two focal strains, the relative fitness of the Gm^R^Kn^R^ strain remained not significantly different from 1 (0.998 ± 0.071, *P* = 0.945) in the absence of kanamycin, suggesting that there was no immediate cost connected to the second resistance gene (Fig. 1B). A relative fitness of 1.212 ± 0.096, significantly higher than 1 (*P* < 0.001) at kanamycin 2.5 μg mL^−1^, suggested positive selection of kanamycin for the Gm^R^Kn^R^ strain. Again, gentamicin had no effect on the relative fitness whether combined with kanamycin or not (*P* > 0.05) (Fig. 1B). This demonstrated that kanamycin at 2.5 μg mL^−1^ had an effect on the growth rate of the Gm^R^ strain, and hence favored the Gm^R^Kn^R^ strain in the competition assay. Gentamicin at all concentrations had no effect on the growth rate or competition of these two focal strains. Consequently, these concentrations were used for testing the set-out hypothesis.

### Effects of antibiotic exposure on the wastewater community’s activity and diversity

**Fig. 2:**
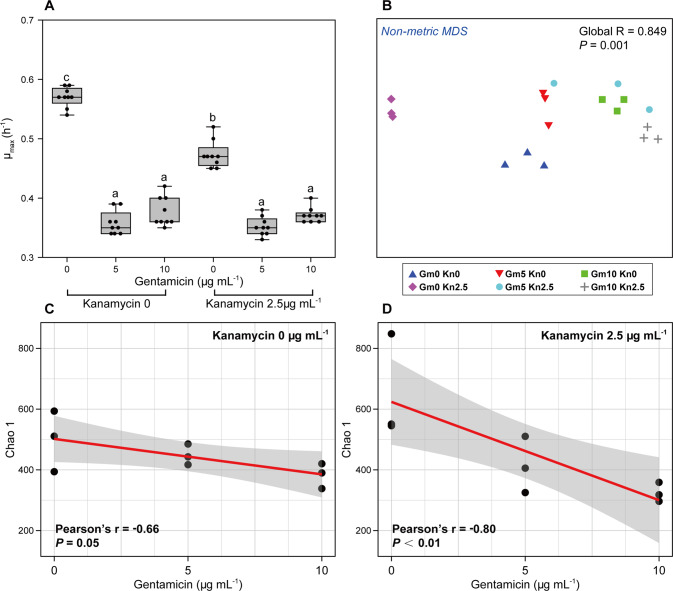
The impact of antibiotics on the wastewater community. **A** Maximum growth rate per hour of the wastewater community across the gradient of gentamicin and kanamycin concentrations. Values are mean ± standard deviation. Significant differences within groups are indicated with different letters (*P* < 0.05). **B** Non-metric multidimensional scaling ordination of the wastewater community composition based on Bray–Curtis similarities. Global r and *P* values are derived from analysis of similarity (ANOSIM). **C** Correlation between Chao 1 richness of the community and gentamicin concentration in the absence of kanamycin and **D** at kanamycin 2.5 μg mL^−1^.

To elucidate the effect of community context on the competition between the two focal strains across antibiotic combinations, the impact of the relevant antibiotics (at the concentrations determined above) on the activity, diversity and composition of the wastewater community need to first be determined. Such potential changes of the wastewater community may alter its effect on the outcome of the competition experiments. The maximum growth rate of the wastewater community as a whole was significantly decreased by gentamicin at 5 μg mL^−1^, from 0.57 ± 0.02 (mean ± SD) in the absence of antibiotics to 0.36 ± 0.02 h^−1^ (*P* < 0.001, ANOVA) (Fig. 2A). Gentamicin at higher concentration or in combination with kanamycin had similar effects (all *P* < 0.05) (Fig. 2A). Kanamycin significantly decreased the growth rate of the wastewater community from 0.57 ± 0.02 to 0.47 ± 0.02 h^−1^ (*P* < 0.001), but to a significantly lesser extent than gentamicin (Fig. 2A). The combination of the two antibiotics decreased the growth rate to a similar degree than gentamicin alone (e.g., 0.37 ± 0.01 h^−1^ at kanamycin 2.5 μg mL^−1^ + gentamicin 10 μg mL^−1^). Consequently, the activity of the wastewater community as a whole was significantly decreased by antibiotic exposure. However, it remains important to note that such whole community-based growth rates integrate the individual growth rates of all individual community members, hence effects of antibiotic exposure on the growth rate of individual members could differ from the here described effects.

The composition of the wastewater communities was also significantly shifted by antibiotic exposure based on non-metric multidimensional scaling (Global R = 0.849, *P* < 0.001, ANOSIM). Communities exposed to high antibiotic concentrations were grouping significantly apart from communities at low or no antibiotic exposure (Fig. 2B). Exposure to only kanamycin and only gentamicin resulted in distinct clusters from each other as well as from the non-antibiotic control treatment (all *P* < 0.01). In the experiments where both antibiotics were present the communities grouped with those under only gentamicin pressure (*P* = 0.07), but different from those only exposed to kanamycin (*P* < 0.01). Consequently, for both, community activity inhibition and diversity, antibiotic exposure displayed a significant effect compared to the control treatment. Gentamicin, which is more potent and was used at higher concentrations, had throughout the stronger effect of the two antibiotics.

However, despite effects on diversity the dominant species in the community did not change across treatments. *Providencia* spp. and *Myroides* spp. dominated the wastewater community; still kanamycin and gentamicin slightly altered the proportion of these observed dominant species (Supplementary Figs. S1 and S2). Previous studies reported that *Providencia* species, at least those isolated from clinical settings, are regularly resistant to aminoglycosides, but more commonly to gentamicin than kanamycin [42, 43]. As gentamicin was in our experiments the more potent drug, this can explain their predominance across all treatments. *Myroides* spp. on the other hand are rarely chromosomally resistant to aminoglycosides. When exploring 17 whole genome sequences of *Myroides* spp. isolates deposited in the NCBI database (Supplementary Table S1), no kanamycin or gentamicin resistance genes were detected. However, 2 of the 17 isolates contained the streptomycin resistance gene *aadS*. The predominance of *Myroides* spp. could however be connected to plasmid encoded resistance, which has previously been described to be rare but possible [44]. Isolation of these two dominant strains to explore their individual properties and effects on selection in depth was attempted but remained unsuccessful.

Among the remaining four species commonly observed at above 1% relative abundance in the non-antibiotic control treatment an unclassified *Enterobacteriaceae* spp. followed a similar trend to *Providencia* and *Myroides*. Contrary, *Salmonella* spp., *Acinetobacter* spp. and *Stenotrophomonas* spp. were all significantly inhibited by gentamicin. While *Salmonella* spp. and *Acinetobacter* spp. where completely inhibited by any gentamicin concentration when compared to the control (all *P* < 0.01) (Supplementary Fig. S2), *Stenotrophomonas* spp. growth was affected by antibiotic exposure but still significantly increased in abundance for all treatments except that containing the highest concentration of both kanamycin and gentamicin (Supplementary Fig. S2).

The Chao 1 richness of the community which also includes the rarer community members was negatively correlated with the gentamicin concentration in both the presence and absence of kanamycin, demonstrating a loss in species diversity. The negative correlation was stronger at 2.5 μg mL^−1^ kanamycin (r = −0.80, *P* < 0.01, Pearson) than in the absence of kanamycin (r = −0.66, *P* = 0.05, Pearson) (Fig. 2C, D). Kanamycin itself did not significantly decrease the richness of the community (*P* = 0.775, ANOVA) (Supplementary Fig. S3). Overall, both gentamicin and kanamycin decreased the activity and altered the composition of the wastewater community. Gentamicin had a greater effect on the activity, diversity and richness of the wastewater community than kanamycin.

### Community context reduces selection for the resistant strain

We aimed to test the hypothesis that community context negatively affects the selection for multidrug resistance, while the presence of a second antibiotic will alleviate this effect. Therefore, the single (Gm^R^) and multidrug (Gm^R^Kn^R^) resistant strains were competed in the presence of the wastewater community across the same antibiotic concentrations as in the competition experiment in the absence of the community. The absolute density of *E. coli* at the end of the experiment was significantly decreased (*P* < 0.01 for all treatments) (Supplementary Fig. S4A) in the presence of the wastewater community compared to the absence of the community, which was expected given that *E. coli* could utilize all available resources in the absence and experienced competition for resources in the presence of the community. However, among those competition experiments in presence of the community the absolute abundance of either *E. coli*, or the wastewater community remained statistically unaltered across all treatments (all *P* > 0.05) (Supplementary Fig. S4B), which indicated that any potential effects would be based on the community composition rather than the communities’ densities.

**Fig. 3:**
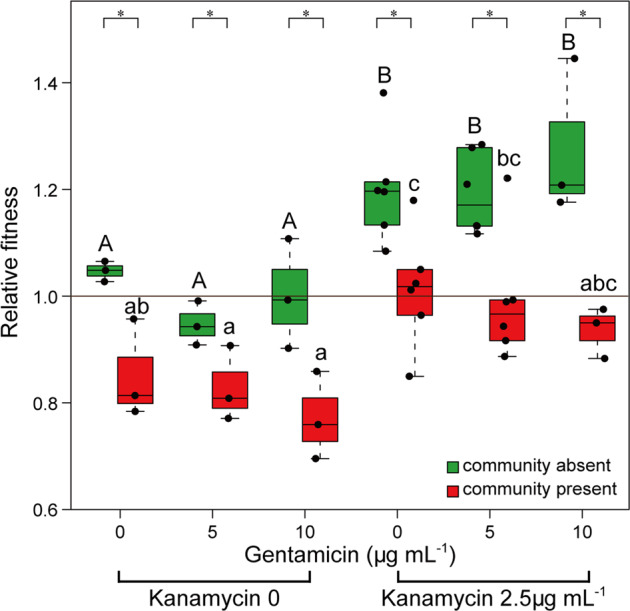
Community context affects the fitness of the Gm^R^Kn^R^ strain. Relative fitness of the Gm^R^Kn^R^ strain in the absence (black) and presence (red) of the community. Values are mean ± standard deviation. Significant differences between groups are indicated with stars. * = *P* < 0.05. Significant differences within groups are indicated with different letters (*P* < 0.05), there were no significant differences between the samples that have the same letters, uppercase and lowercase represent different groups.

In the presence of the wastewater community, the relative fitness of the Gm^R^Kn^R^ strain compared to the Gm^R^ strain was significantly lower than in the absence of the wastewater community (*P* < 0.05, ANOVA) (Fig. 3). The relative fitness was significantly lower than 1 in the absence of antibiotics (0.852 ± 0.093, mean ± SD, *P* = 0.020), suggesting that the wastewater community imposed an additional cost on the Gm^R^Kn^R^ strain (Fig. 3). Although the presence of the antibiotic kanamycin at 2.5 µg mL^−1^ positively selected the Gm^R^Kn^R^ strain in the absence of the wastewater community, the presence of the wastewater community significantly reduced this positive selection through kanamycin, from 1.212 ± 0.096 to 1.013 ± 0.108 (*P* < 0.001), which accounts for only neutral selection not significantly different from 1 (*P* = 0.773) (Fig. 3).

Similar to the competition experiment in the absence of the wastewater community, gentamicin had no effect on the relative fitness of the Gm^R^Kn^R^ strain in the presence of the wastewater community (*P* > 0.05). This was in spite of the significant decreases in activity and richness observed for the wastewater community in the presence of gentamicin (Fig. 3).

To sum up, the reduction in relative fitness of the Gm^R^Kn^R^ strain in presence of the community was consistent across gentamicin concentrations, despite gentamicin reducing the activity and richness of the community.

### Inhibitory effects of antibiotics were maintained during the incubation period

**Fig. 4:**
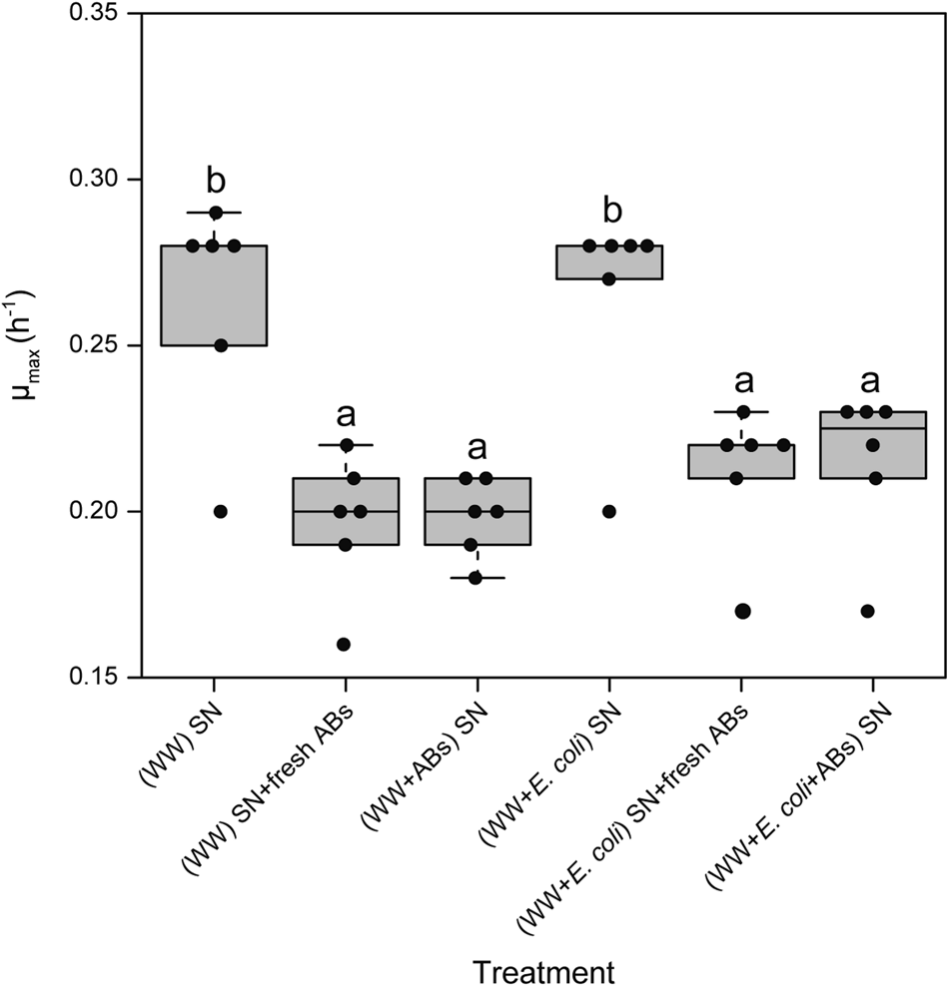
Maximum growth rate per hour of the Gm^R^ strain at different treatments. Values are mean ± standard deviation. (WW) SN: supernatant of the wastewater community culture. (WW) SN + fresh ABs: supernatant of the wastewater community culture with fresh antibiotics added. (WW + ABs) SN: supernatant of the wastewater community culture in the presence of antibiotics. (WW + *E. coli*) SN: supernatant of the focal strains and wastewater community culture. (WW + *E. coli*) SN + fresh ABs: supernatant of the focal strains and wastewater community culture with fresh antibiotics added. (WW + *E. coli* + ABs) SN: supernatant of the focal strains and the wastewater community culture in the presence of antibiotics. The antibiotics were added at gentamicin 10 µg mL^−1^ and kanamycin 2.5 µg mL^−1^. Significant differences are indicated with different letters (*P* < 0.05, ANOVA).

To ensure that the observed effects were not based on the degradation of the used antibiotics through the wastewater community, we tested the maximum growth rate of the Gm^R^ strain in different supernatants of the experiments. All treatments containing antibiotics displayed a significantly decreased maximum growth rate of the Gm^R^ strain compared to supernatant from the non-antibiotic control treatment (all *P* < 0.05) (Fig. 4). The maximum growth rate in supernatant of the antibiotic-free treatment with fresh antibiotics added (Gm 10 µg mL^−1^ + Kn 2.5 µg mL^−1^) (0.20 ± 0.02, mean ± SD) was similar to that in supernatant of the treatment in the presence of same concentrations of antibiotics (0.21 ± 0.02). This was true for supernatants of both, the pure wastewater community without (*P* = 0.867, ANOVA) and the community grown together with the focal strains (*P* = 0.802) (Fig. 4). These results indicated that the antibiotics were indeed not degraded by bacteria, neither the wastewater community, nor the focal strains, and that hence the inhibitory effects of antibiotics persisted during the three-day incubation period.

### Confirmation of the observed effects based on a second combination of antibiotics and resistance genes

To explore whether the results above are applicable to other combinations of antibiotics, a single kanamycin resistant (Kn^R^) strain and a multidrug kanamycin and streptomycin resistant strain (Kn^R^Sp^R^) were similarly competed in the absence and presence of the wastewater community across combinations of two streptomycin and three kanamycin concentrations. Similarly, in the individual growth rate assay, streptomycin significantly decreased the maximum growth rate of the Kn^R^ strain by 64.2 ± 5.2 % (mean ± SD, *P* < 0.001, ANOVA) (Supplementary Fig. S5A). However, streptomycin had no effect on the maximum growth rate of the Kn^R^Sp^R^ strain (*P* = 0.498) (Supplementary Fig. S5A). Kanamycin had no impact on both strains and the effect of combinations of kanamycin and streptomycin did not significantly differ from streptomycin alone for either strain (*P* > 0.05) (Supplementary Fig. S5A).

In the competition experiment between the focal strains in absence of the community, the relative fitness of the Kn^R^Sp^R^ compared to the Kn^R^ strain remained not significantly different from 1 (1.05 ± 0.135, *P* = 0.364) in the absence of antibiotics (Supplementary Fig. S5B), suggesting there was again no fitness cost of the second resistant gene. The relative fitness at streptomycin 25 μg mL^−1^ was significantly higher than 1 (1.262 ± 0.135, *P* < 0.001) (Supplementary Fig. S5B), indicating positive selection through streptomycin on the Kn^R^Sp^R^ strain. Again, kanamycin, which both strains possess resistance to, had no impact on the relative fitness throughout (*P* > 0.05) (Supplementary Fig. S5B).

With regards to the effect of the antibiotics on the wastewater community, streptomycin at 25 μg mL^−1^ significantly decreased the maximum growth of the wastewater community from 0.52 ± 0.05 to 0.42 ± 0.01 (*P* < 0.001) (Supplementary Fig. S6). Moreover, kanamycin had a stronger effect on the maximum growth rate of the wastewater community than streptomycin, the maximum growth rates at both kanamycin 12.5 and 25 μg mL^−1^ (0.30 ± 0.02) were significantly lower than those at streptomycin 25 μg mL^−1^ (*P* < 0.001) (Supplementary Fig. S6). The combination of the two antibiotics had a significantly stronger effect than any single antibiotic on the maximum growth rate of the wastewater community (0.22 ± 0.02) (*P* < 0.001) (Supplementary Fig. S6).

In the presence of the wastewater community, the relative fitness of the Kn^R^Sp^R^ strain was significantly decreased in the absence of antibiotics (*P* < 0.05). Here, the relative fitness was significantly lower than 1 (0.868 ± 0.101, *P* < 0.001) (Supplementary Fig. S7). Furthermore, the wastewater community significantly decreased the relative fitness at streptomycin 25 μg mL^−1^, from 1.262 ± 0.135 to 1.020 ± 0.055 (*P* < 0.05), indicating that the positive selection of streptomycin was eliminated by the wastewater community (Supplementary Fig. S7). Again, this effect could not be overcome by the addition of kanamycin, despite its inhibitory effects on the community’s activity (Supplementary Fig. S7).

Overall, the results of the competition experiment of the Kn^R^ and Kn^R^Sp^R^ strains in the absence and presence of the wastewater community followed similar dynamics than those observed for the Gm^R^ and Gm^R^Kn^R^ strains, which confirms that community context-based effects resulting in the reduction in relative fitness of multidrug resistant strains are consistent to antibiotic pressure through additional antibiotics.

## Discussion

In this study, we demonstrate how competition between a multidrug and a single resistant focal strain is affected by the presence of a model wastewater community during exposure to a combination of antibiotics. Consistent results were obtained from different pairwise combinations of different resistance genes: The relative fitness of and thus selection for the multidrug resistant strain was significantly decreased in the presence of the wastewater community, indicating that community context imposed a cost on the multidrug resistant strain. Contrary to our hypothesis, reduced selection through community context was consistent under pressure of a second antibiotic, even though both the activity and the diversity of the community were significantly reduced.

Previous studies reported that the presence of other interacting, potentially competing microbes can indeed significantly affect the selection for resistance in a focal bacterial strain under antibiotic pressure [19, 45]. Here, the maintenance of a second, originally cost-neutral resistance gene imposed a significant cost on the multidrug compared to the single resistant strain when in community context in the absence of selection pressure. The community-imposed fitness costs for this single additional resistance gene were small, likely allowing persistence in the environment [46]. Still, when amplifying such fitness costs by multiple resistance genes within one bacterial strain, community context could, in the absence of selection, provide a natural barrier to the dissemination of multidrug resistance [15]. The proposed underlying mechanisms included an increase in costs of resistance when in competition with the community as well as a protective effect towards the susceptible strain through the community [19]. Interspecies interactions can protect the susceptible bacteria in three main ways, collective resistance, collective tolerance and exposure protection [45, 47, 48]. The underlying mechanisms for this include cooperative inactivation of antibiotics, competitive interactions and biofilm formation [45]. In this study, cooperative detoxification of the environment from antibiotics by resistant cells in the community [49] may, if at all, only play a minor role in the observed protection effect through community context. As we demonstrate that the full antibiotic effect on the susceptible strain could still be observed from supernatants after the incubation period during which degradation would have taken place. Still, microniches with low antibiotic concentrations could have transitionally existed in the direct proximity of such resistant community members. However, antibiotic concentrations would have quickly equilibrated through continuous shaking at high speeds. Due to this continuous shaking throughout the experiments, biofilm formation was also not visually observed and biofilm induced exposure protection may consequently not represent the mechanism underlying the dynamics observed in this study. Hence, competitive interactions with other community members, the main type of interaction among bacterial species in complex communities [50, 51], are suggested to play the most important role in reducing the fitness of the resistant strain, mainly by increasing the cost of the additionally carried resistance gene [19].

Here, independent of the choice of antibiotics, reduced selection for the multidrug resistant strain in the community context was consistent with pressure by the second antibiotic, even though the second antibiotic significantly decreased the activity and altered the composition of the community. This indicated that the community effect on selection was not dependent on the activity or competitive ability of the entire community which can cause increased nutrition and niche availability for the focal strain [52], but rather on immediate competition with a small proportion of bacteria within the community. Indeed, the highest degree of competition for the focal species is usually imposed through those members of a community with a high degree of niche overlap compared to the focal strain [53]. If these high-level competitor bacteria within the community are tolerant to the antibiotic effects, the community effect on selection is consistent. Antibiotic effects can here refer to direct inhibitory effects, but also include indirect ones through changes in community structure and metabolic networks [54, 55]. In our experiments, this is likely the case, as for example the dominant species in the community did not change across all antibiotic concentrations. This indicates that indeed strongly tolerant community members, exist. One such example is the most dominant observed species *Providencia* spp., which has been regularly reported as aminoglycoside resistant [42, 43]. Pulsed exposure to the antibiotic in question could further increase the community’s competitive ability, especially if resistance in the focal strain is encoded on mobile genetic elements and can be obtained by highly competitive community members [56]. While in this study the resistance gene is chromosomally encoded, transferability could hence have additional effects on reduced selection for resistance in the focal strain. Future studies using artificial, well-characterized assembled communities could reveal the individual contributions of general competition with all community members through e.g., resource or spatial limitations [57] versus high-level competitors within the community with overlapping niches to the focal strain on selection for antibiotic resistance.

An additional aspect to consider with regards to selection in the presence of multiple antibiotics are the interactions between the antibiotics themselves, where their potency could be altered when used in combination through synergistic or antagonistic effects [58, 59]. Such effects are of high importance when aiming to reduce or inhibit multidrug resistant bacteria [29]. Further, acquiring resistance genes towards one antibiotic could result in cross-resistance or collateral sensitivity to other antibiotics [30]. However, in this study the resistance genes introduced to the focal strains did not confer either cross-resistance or collateral sensitivity, even if all tested antibiotics belonged to the same antibiotic class of aminoglycosides. In addition, the level of inhibition of the community for combinations of the antibiotics was throughout higher or identical to the strongest effect of a single antibiotic, hence excluding antagonistic effects canceling out effects of the second antibiotic, which could have led to the observed dynamics.

In summary, we have shown that reduced selection for antibiotic resistance in a community context can be consistent to pressure by additional antibiotics. This effect likely stems from an increased cost of resistance imposed through competition with certain individual community members that maintain their high-level competitive ability under antibiotic pressure, despite severe effects of the antibiotics on community activity and structure. The concentrations of the additional antibiotic used in this study were above those usually expected in the environment [21], indicating that at environmentally relevant concentrations reduced selection for antibiotic resistance through community context will be widely unaffected by the presence of additional stressors. The ability of communities to amplify the fitness costs of resistance genes, even under conditions where certain community members and functions are impaired could hence provide an important natural barrier to the dissemination of multidrug resistance.

## Supplementary information

Supplementary Materials

## Data Availability

All data is available in the main text or Supplementary materials. All sequences in this study were deposited in the NCBI Sequence Read Archive (SRA) database under project number PRJNA865074.
