# Supplementary material for: Reduced selection for antibiotic resistance in community context is maintained despite pressure by additional antibiotics
Source: ISME Commun. 2023 May 31;3:52. doi: 10.1038/s43705-023-00262-4 (PMC10232432; doi:10.1038/s43705-023-00262-4)
Supplement: Supplementary file 1 — Supplementary Materials [file 43705_2023_262_MOESM1_ESM.docx]

*Supplementary information of the article:*

Reduced selection for antibiotic resistance in community context is maintained despite pressure by additional antibiotics

**Author list**

Peiju Fang^1^, Alan Xavier Elena^1^, Maxi Antonia Kunath^1^, Thomas U. Berendonk^1^, Uli Klümper^1,#^

^1^ Technische Universität Dresden, Institute of Hydrobiology, Dresden, Zellescher Weg 40, Germany

^#^ Corresponding author:

Dr. Uli Klümper (ORCID: 0000-0002-4169-6548)

Technische Universität Dresden, Institute of Hydrobiology,

01062 Dresden,

Zellescher Weg 40,

Germany

Phone: (+49) 351 46343273

E-mail: [Uli.Kluemper@tu-dresden.de](mailto:Uli.Kluemper@tu-dresden.de)

**This supplementary information contains:**

- 9 Pages
- 7 Figures
- 1 Table


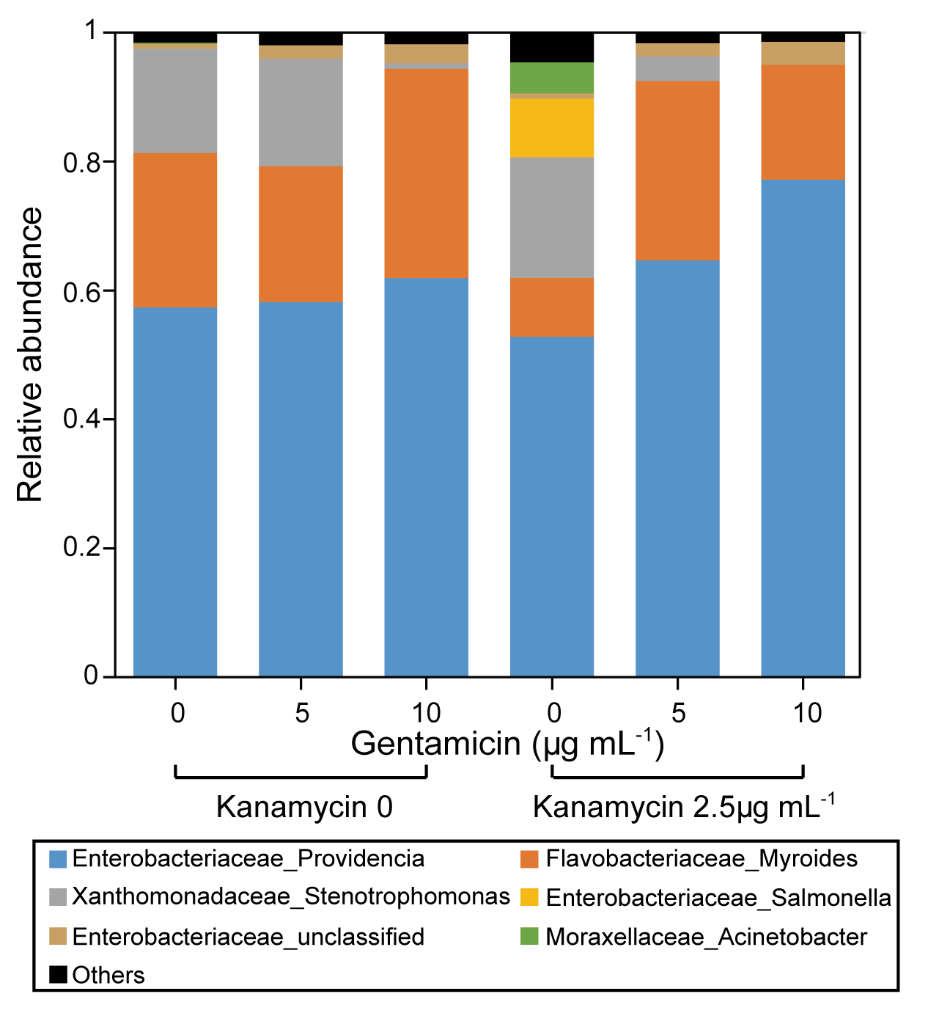


**Fig. S1.** Composition of the bacterial wastewater community at the end of the competition experiments. Relative abundance of the dominant OTUs across the different antibiotic concentration. Values refer to the average OTU abundance of three replicates. Others: OTUs with a relative abundance below 1%.


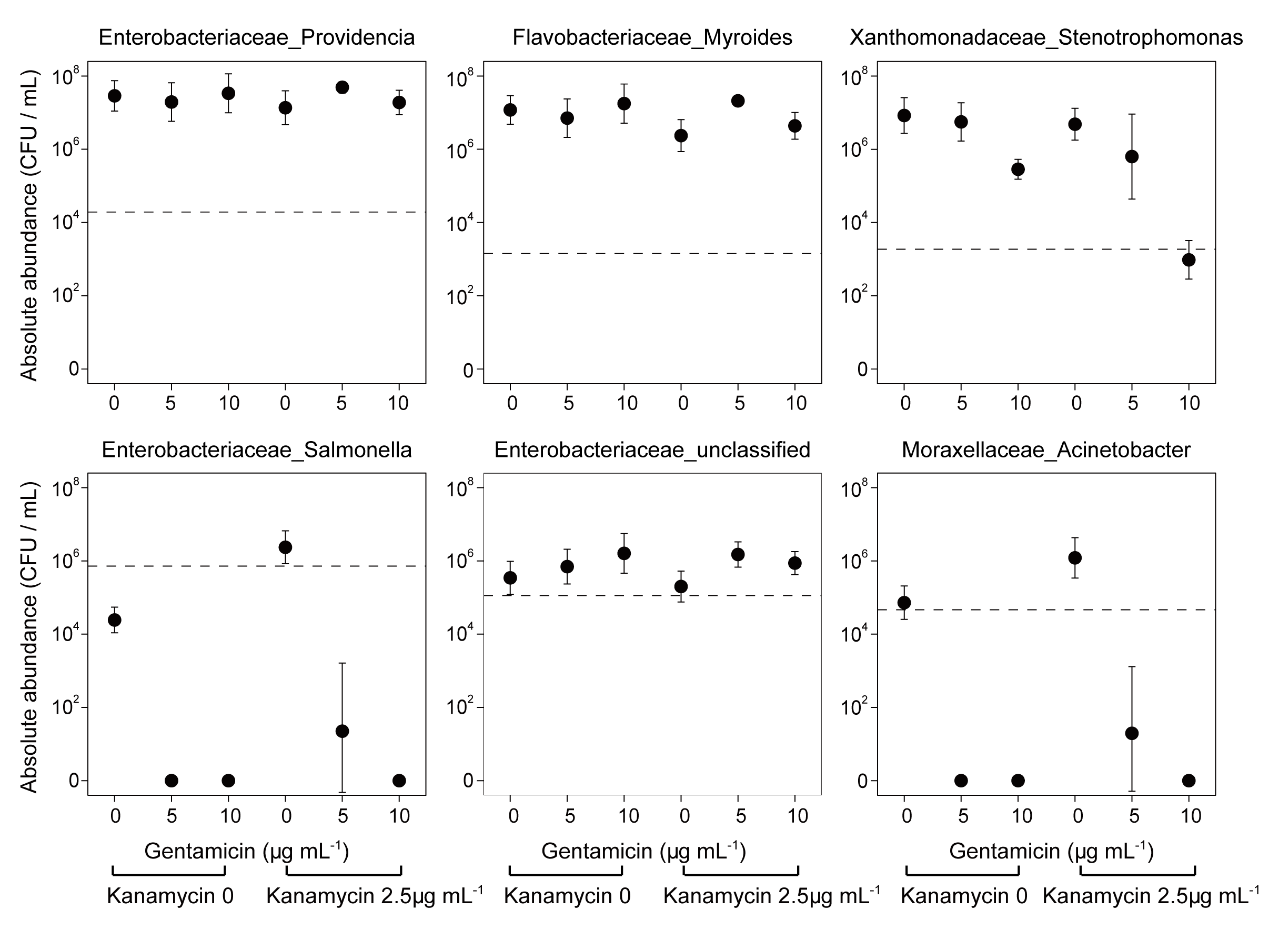


**Fig. S2.** Absolute abundance of the dominant OTUs in the wastewater across antibiotic concentrations. The dashed lines refer to the initial inoculate concentration.


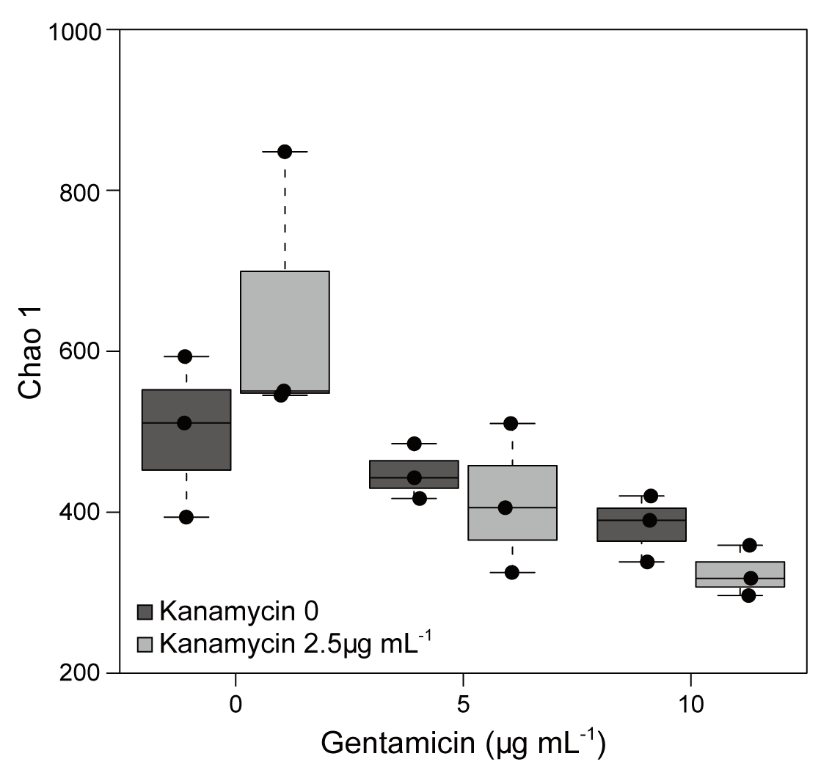


**Fig. S3.** Kanamycin did not alter the richness of the wastewater community. Pairwise comparisons of Chao 1 richness of the wastewater community at kanamycin 0 and 2.5 μg mL^-1^ across gentamicin concentrations were not significant throughout (all *P* > 0.05).


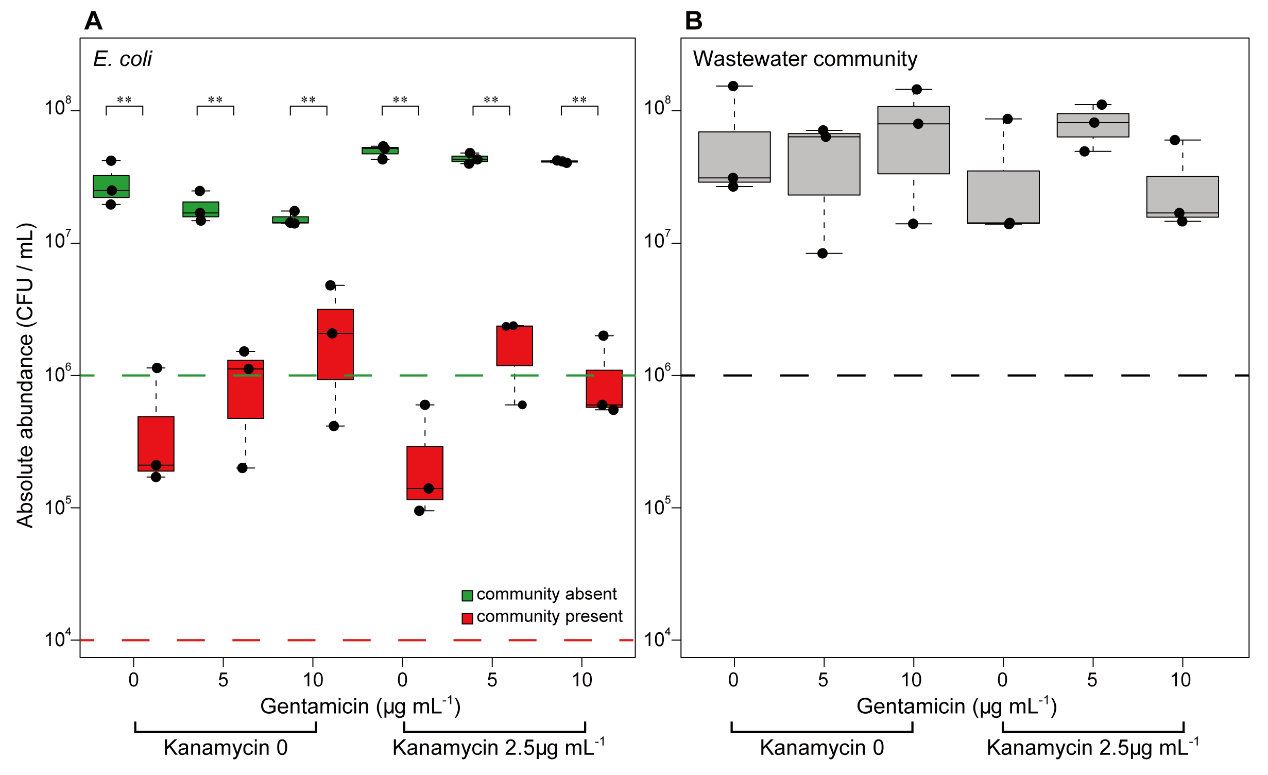


**Fig. S4.** Absolute bacterial abundance of the focal *E. coli* **(A)** and the wastewater community **(B)** at the end of the competition experiments. Dashed lines refer to the initial inoculate concentration at the start of the experiment. Significant differences between groups are indicated with stars. **, *P* < 0.01.


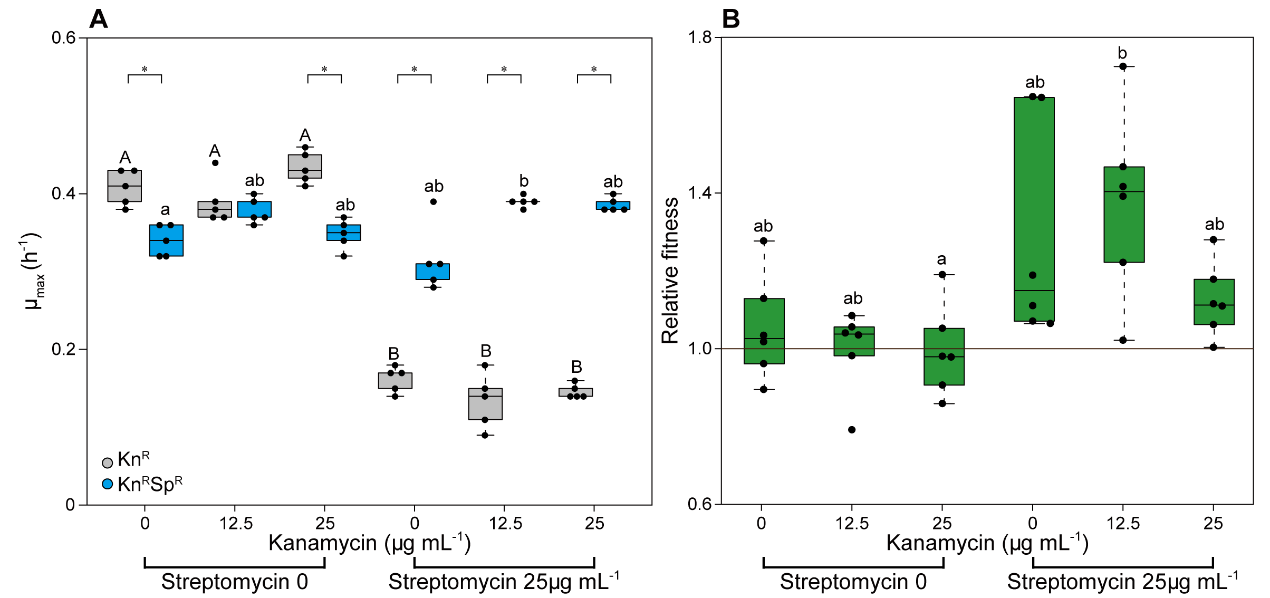


**Fig. S5.** Streptomycin decreased the activity of the Kn^R^ strain. **(A)** The maximum growth rate per hour of the Kn^R^ and Kn^R^Sp^R^ strains across the gradient of kanamycin and streptomycin concentration. **(B)** Relative fitness of Kn^R^Sp^R^ strain vs Kn^R^ strain. Values are mean ± standard deviation. Significant differences between groups are indicated with stars (*P* < 0.05). *, *P* < 0.05. Significant differences within groups are indicated with different letters (*P* < 0.05), there were no significant differences between the samples that have the same letters, uppercase and lowercase represent different groups.


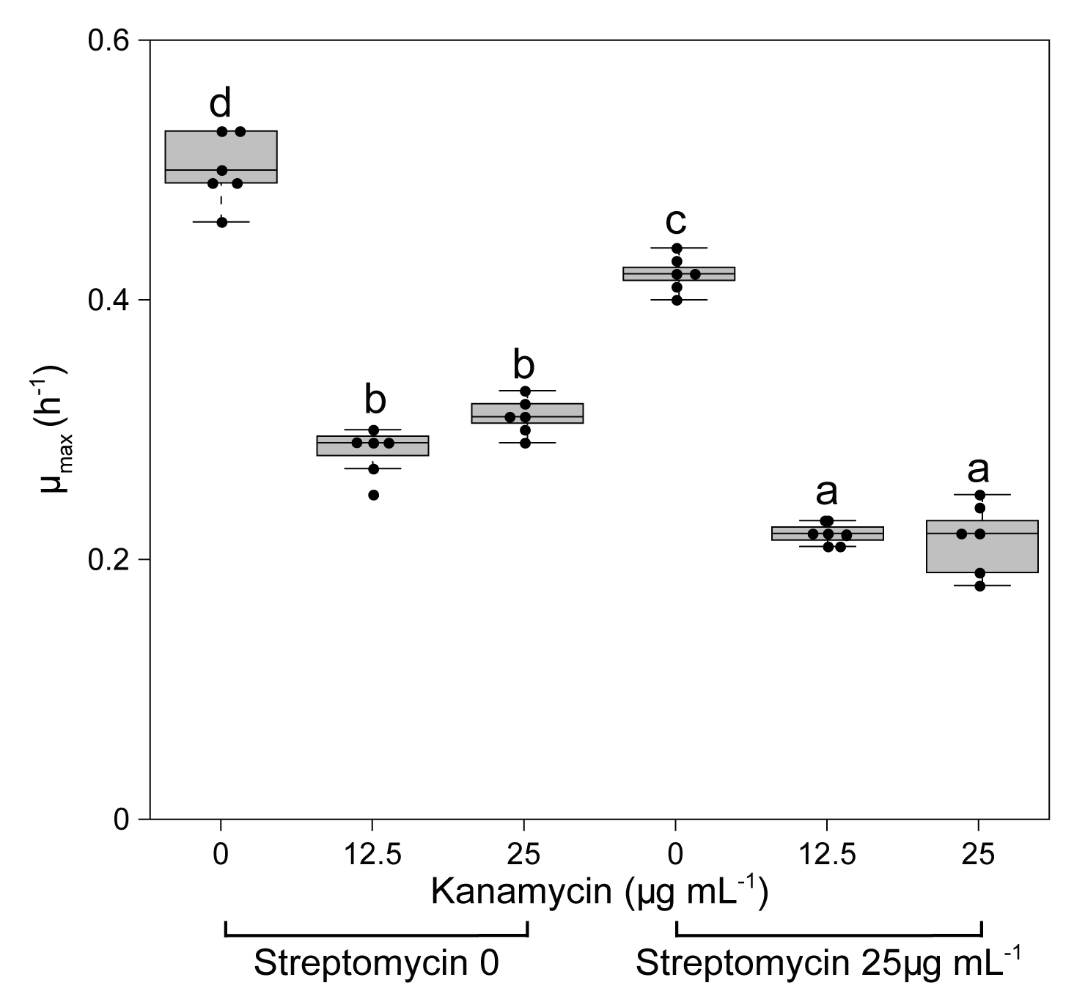


**Fig. S6.** Activity of the wastewater community is decreased under antibiotic pressure. Maximum growth rate per hour of the wastewater community across the gradient of kanamycin and streptomycin concentrations. Values are mean ± standard deviation. Significant differences within groups are indicated with different letters (*P* < 0.05).


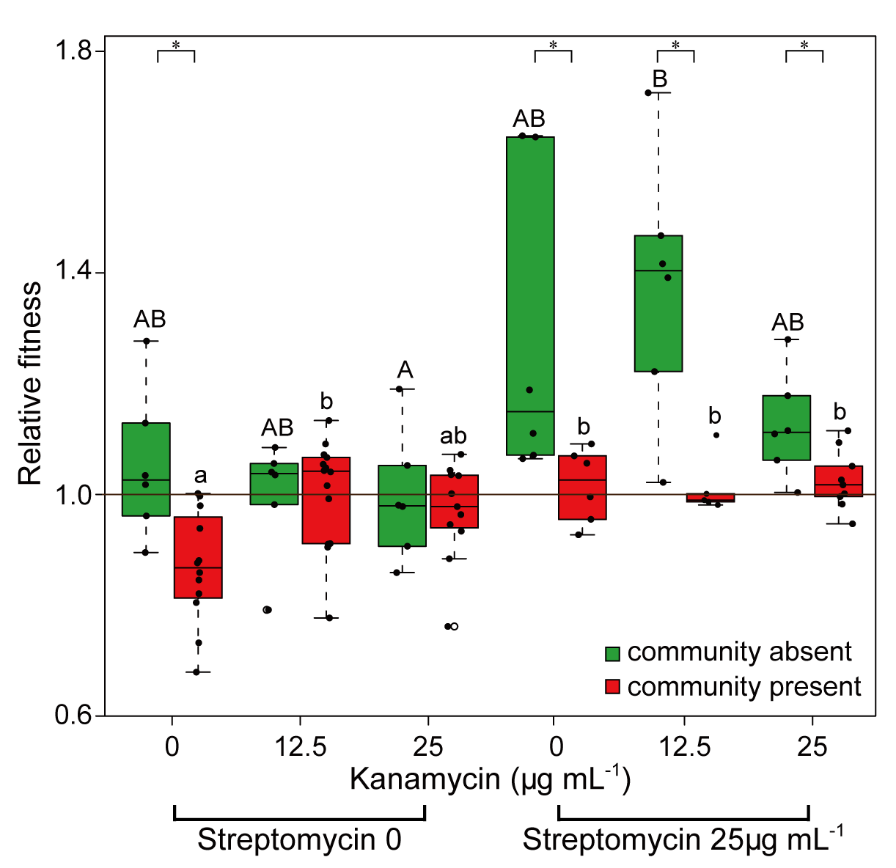


**Fig. S7.** Relative fitness of Kn^R^Sp^R^ strain vs Kn^R^ strain. Values (mean ± standard deviation) in the absence (black) and presence (red) of the community. Significant differences between groups are indicated with stars (*P* < 0.05). *, *P* < 0.05. Significant differences within groups are indicated with different letters (*P* < 0.05), there were no significant differences between the samples that have the same letters, uppercase and lowercase represent different groups.**Table S1.** Predicted aminoglycoside resistance genes in 17 whole-genome-sequenced *Myroides* spp. isolates downloaded from the NCBI Genbank database. “-“ = no ARG present.

|  | Isolate | Gentamicin  ARGs | Kanamycin  ARGs | Streptomycin  ARGs |
| --- | --- | --- | --- | --- |
| 1 | CP013690 | _ | _ | *aadS* |
| 2 | CP037427 | _ | _ | _ |
| 3 | JH590834 | _ | _ | *aadS* |
| 4 | JH590837 | _ | _ | _ |
| 5 | JH815535 | _ | _ | _ |
| 6 | KE161015 | _ | _ | _ |
| 7 | KE340319 | _ | _ | _ |
| 8 | UGQP01000001 | _ | _ | _ |
| 9 | CM001437 | _ | _ | _ |
| 10 | CP068107 | _ | _ | _ |
| 11 | LROZ01000001 | _ | _ | _ |
| 12 | CP068107 | _ | _ | _ |
| 13 | JANUEE010000001 | _ | _ | _ |
| 14 | JH815539 | _ | _ | _ |
| 15 | LROZ01000001 | _ | _ | _ |
| 16 | UGQL01000001 | _ | _ | _ |
| 17 | UGQN01000001 | _ | _ | _ |
